# Pharmacodynamic Evaluation of Dosing, Bacterial Kill, and Resistance Suppression for Zoliflodacin Against *Neisseria gonorrhoeae* in a Dynamic Hollow Fiber Infection Model

**DOI:** 10.3389/fphar.2021.682135

**Published:** 2021-05-21

**Authors:** Susanne Jacobsson, Daniel Golparian, Joakim Oxelbark, Emilie Alirol, Francois Franceschi, Tomas N. Gustafsson, David Brown, Arnold Louie, George Drusano, Magnus Unemo

**Affiliations:** ^1^ WHO Collaborating Centre for Gonorrhoea and Other STIs, National Reference Laboratory for Sexually Transmitted Infections, Department of Laboratory Medicine, Faculty of Medicine and Health, Örebro University, Örebro, Sweden; ^2^ Division of Clinical Chemistry, Department of Laboratory Medicine, Faculty of Medicine and Health, Örebro University, Örebro, Sweden; ^3^ Global Antibiotic Research and Development Partnership (GARDP), Geneva, Switzerland; ^4^ Department of Clinical Microbiology, Sunderby Research Unit, Umeå University, Umeå, Sweden; ^5^ Institute for Therapeutic Innovation, College of Medicine, University of Florida, Orlando, FL, United States

**Keywords:** *Neisseria gonorrhoeae*, hollow fiber infection model, zoliflodacin, pharmacodynamics, antimicrobial treatment, pharmacokinetics

## Abstract

Antimicrobial resistance in *Neisseria gonorrhoeae* is threatening the treatment and control of gonorrhea globally, and new treatment options are imperative. Utilizing our dynamic *in vitro* hollow fiber infection model (HFIM), we examined the pharmacodynamics of the first-in-class spiropyrimidinetrione (DNA gyrase B inhibitors), zoliflodacin, against the *N. gonorrhoeae* reference strains World Health Organization F (susceptible to all relevant antimicrobials) and WHO X (extensively drug resistant, including resistance to ceftriaxone) over 7 days. Dose-range experiments with both strains, simulating zoliflodacin single oral dose regimens of 0.5–8 g, and dose-fractionation experiments with WHO X, simulating zoliflodacin oral dose therapy with 1–4 g administered as q12 h and q8 h for 24 h, were performed. A kill-rate constant that reflected a rapid bacterial kill during the first 6.5 h for both strains and all zoliflodacin doses was identified. In the dose-range experiments, the zoliflodacin 2–8 g single-dose treatments successfully eradicated both WHO strains, and resistance to zoliflodacin was not observed. However, zoliflodacin as a single 0.5 g dose failed to eradicate both WHO strains, and a 1 g single dose failed to eradicate WHO X in one of two experiments. The zoliflodacin 1 g/day regimen also failed to eradicate WHO X when administered as two and three divided doses given at q12 h and q8 h in the dose-fractionation studies, respectively. All failed regimens selected for zoliflodacin-resistant mutants. In conclusion, these data demonstrate that zoliflodacin should be administered at >2 g as a single oral dose to provide effective killing and resistance suppression of *N. gonorrhoeae*. Future studies providing pharmacokinetic data for zoliflodacin (and other gonorrhea therapeutic antimicrobials) in urogenital and extragenital infection sites, particularly in the pharynx, and evaluation of gonococcal strains with different *gyrB* mutations would be important.

## Introduction

In 2016, *Neisseria gonorrhoeae* was estimated to cause 87 million new global gonorrhea cases among adults each year (Rowley et al., 2016), an increase of 12% compared to the 78 million estimated cases in 2012 (Newman et al., 2015). In addition to the increasing incidence internationally, the emergence and spread of antimicrobial resistance (AMR) in *N. gonorrhoeae* globally seriously threatens the management and control of gonorrhea (Wi et al., 2017; Day et al., 2018; Unemo et al., 2019). This development prompted the World Health Organization (WHO) and the Centers for Disease Control and Prevention (CDC) to categorize *N. gonorrhoeae* as a priority 2 (high) pathogen and an urgent threat, respectively (WHO, 2017; CDC, 2019). As strongly emphasized in the WHO Global Action Plan to control the spread and impact of antimicrobial resistance in *N. gonorrhoeae* (WHO, 2012) and the WHO Global Action Plan on antimicrobial resistance (WHO, 2015), new treatment options are urgently needed for *N. gonorrhoeae*, and research and development efforts for novel antibiotics for the treatment of urogenital and extragenital gonorrhea are of the highest priority.

Zoliflodacin represents the first drug in a novel class of type II topoisomerase inhibitors called spiropyrimidinetriones. Zoliflodacin has a unique mode of action with the binding site in the GyrB subunit of the bacterial DNA gyrase that is distinct from binding sites of fluoroquinolones that target GyrA and ParC. Previous studies have shown that zoliflodacin is bactericidal, with a low frequency of resistance, and potent *in vitro* activity against *N. gonorrhoeae*, including multidrug-resistant and extensively drug-resistant (XDR) strains (minimum inhibitory concentrations (MICs) of zoliflodacin ranging from ≤0.002 to 0.25 mg/L) (Alm et al., 2015; Basarab et al., 2015; Foerster et al., 2015; Foerster et al., 2019; Bradford et al., 2020; Unemo et al., 2021). So far, no clinical zoliflodacin-resistant *N. gonorrhoeae* isolates or clinical *N. gonorrhoeae* strains with zoliflodacin resistance mutations in the target, GyrB, have been found (Bradford et al., 2020). However, zoliflodacin-resistant mutants have been selected *in vitro*: all containing nonsynonymous mutations in amino acid codons D429 or K450 of GyrB, which were verified to cause the increased MICs of zoliflodacin in the mutants (Alm et al., 2015; Foerster et al., 2015; Foerster et al., 2019). Zoliflodacin has undergone two phase 1 clinical trials. The first trial investigated the safety, tolerability, and pharmacokinetics (PK) of zoliflodacin in both fed and fasted states, and the second focused on determining the absorption, distribution, metabolism, and excretion (ADME). Zoliflodacin showed linear PK, good oral bioavailability, and no significant safety findings (Basarab et al., 2015; O’Donnell et al., 2019). Subsequently, a phase 2 randomized controlled comparative clinical trial (RCT) was conducted to evaluate the efficacy and safety of zoliflodacin 2 and 3 g single oral dose for the treatment of uncomplicated gonorrhea. The microbiological cure rates for urogenital gonorrhea in the per-protocol analyses were 98% (48/49) for zoliflodacin 2 g and 100% (47/47) for zoliflodacin 3 g. The microbiological cure rates for rectal infections were 100% (4/4) and 100% (6/6), and for pharyngeal infections, 67% (4/6) and 78% (7/9), respectively (Taylor et al., 2018). No *N. gonorrhoeae* isolates with *in vitro* resistance to zoliflodacin were found (Taylor et al., 2018). It is not known whether insufficient exposure of zoliflodacin is linked to cure failure at these body sites. Appropriate, systematic PK and pharmacodynamic (PD) evaluations, and antimicrobial PD examinations (integrating microbiology and pharmacology) are required to further understand these findings of the zoliflodacin phase 2 RCT (Taylor et al., 2018) and to optimize zoliflodacin dosing regimens for ideal *N. gonorrhoeae* kill as well as suppression of resistance emergence. Accordingly, an appropriate hollow fiber infection model (HFIM) for *N. gonorrhoeae* has been urgently needed, because previous *in vitro* zoliflodacin studies have only used static time–kill curve models and simplified PD modeling, including many inherent limitations (Foerster et al., 2015; Foerster et al., 2019). Nevertheless, recently an HFIM was used to examine the relationship between gepotidacin exposure and prevention of on-therapy resistance amplification in *N. gonorrhoeae* (VanScoy et al., 2020). Therefore, we have developed, standardized, and quality assured a dynamic *in vitro* HFIM to simulate gonococcal infections and the PK/PD of antimicrobials acting against *N. gonorrhoeae* infections, using geographically, phenotypically, and genomically diverse WHO *N. gonorrhoeae* reference strains (Unemo et al., 2016)*.*

The overarching aim of the present study was to examine the PD of zoliflodacin against *N. gonorrhoeae* in our dynamic *in vitro* HFIM. Specific aims included performing dose-range and dose-fractionation studies in the HFIM to 1) identify the dynamically linked PD indices for zoliflodacin in *N. gonorrhoeae* kill and resistance suppression, 2) determine the dynamic rate of *N. gonorrhoeae* killing with zoliflodacin, and 3) examine optimal zoliflodacin dosing for gonorrhea. The results of the present study not only provide further understanding of the findings of the zoliflodacin phase 2 RCT (Taylor et al., 2018), the concentration-dependent killing of *N. gonorrhoeae* with zoliflodacin (most effective when giving the whole dose once), importance of examining multiple divergent *N. gonorrhoeae* strains, and suppression of AMR emergence but also serve us to inform the initiated zoliflodacin phase 3 RCT (ClinicalTrials.gov identifier NCT03959527) as well as future dosing, registration, and introduction of zoliflodacin in clinical practice for gonorrhea.

## Material and Methods

### Bacterial Strains

The *N. gonorrhoeae* reference strains WHO F (susceptible to all relevant antimicrobials) and WHO X (XDR, including resistance to first-line ceftriaxone) (Unemo et al., 2016) were examined.

### *In Vitro* MIC (mg/L) Determination

For zoliflodacin, agar dilution was performed, according to Clinical and Laboratory Standards Institute (CLSI) guidelines (M07-A9 and M100-S24; www.clsi.org), on GCVIT agar plates (3.6% Difco GC Medium Base agar (BD, Diagnostics, Sparks, MD, United States) supplemented with 1% IsoVitalex (BD, Diagnostics, Sparks, MD, United States). Additionally, a microbroth dilution method for zoliflodacin (in triplicate) was performed in the medium used in the HFIM, that is, modified fastidious broth (mFB), as previously described (Foerster et al., 2020). Etest was used for MIC testing of ceftriaxone, cefixime, ciprofloxacin, and azithromycin, in accordance with the manufacturer’s instructions (bioMérieux, Marcy-l’Etoile, France).

### The Hollow Fiber Infection Model

For simulation of gonococcal infections and the PK/PD of zoliflodacin against *N. gonorrhoeae*, our dynamic *in vitro* HFIM, using mFB (Foerster et al., 2020) and cellulosic HFIM cartridges (FiberCell Systems Inc., Frederick, MD, United States), was used*.* Briefly, the HFIM is a two-compartment model system where the bacteria grow in the extra-capillary space within a cellulosic cartridge containing a bundle of microfibers (FiberCell Systems Inc., Frederick, MD, United States). The antibiotic was administered to the HFIM by a syringe pump, and peristaltic pumps isovolumetrically replaced drug-containing broth medium with drug-free medium to simulate the plasma half-life and free (non–protein-bound fraction) drug concentration–time profiles for zoliflodacin reported in humans throughout the study. Sampling for bacterial quantitative cultures (colony-forming units (CFUs)/mL) for total bacterial burden and possible zoliflodacin-resistant population and measurement of drug concentrations was performed over 7 days (Cadwell, 2012; Drusano, 2017).

On the first day of the experiment, 0.5 ml of fresh *N. gonorrhoeae* cultures (18–24 h) from GCAGP agar plates (Foerster et al., 2019) were inoculated in 49.5 ml of mFB and incubated at 36°C in a humidified 5% CO_2_-enriched atmosphere to mid-log phase. 10 mL (quantified as ∼10^5^–10^6^ CFU/ml in the different experiments) of the bacterial suspensions were inoculated into the extra-capillary space of each HFIM cartridge to simulate a clinically relevant *N. gonorrhoeae* burden in different anatomical sites of infection (Bissessor et al., 2011; Chow et al., 2016; Priest et al., 2017; Van Der Veer et al., 2020). Zoliflodacin was administrated to the HFIM *via* a syringe pump (New Era Pump Systems Inc., Farmingdale, NY, United States) programmed to mimic a fasted adult human protein-unbound PK concentration–time profile following a single oral zoliflodacin dose (PK parameters were used for oral dose of zoliflodacin 3 g (linear PK assumed throughout all doses evaluated): 17% protein-unbound fraction of zoliflodacin in human plasma, 6.47 h half-life (T_1/2_), and a 3-h infusion time) (Study STI_Zoli002; O’Donnell et al., 2019). One HFIM cartridge per examined strain and experiment did not receive any zoliflodacin and served as an untreated growth control.

A series of zoliflodacin dose-range and dose-fractionation experiments were conducted. For the dose-range experiments (*n* = 2), simulated single dose oral therapy for zoliflodacin with dosages of 0.5, 1, 2, 3, 4, 6, and 8 g was tested against the WHO F and WHO X reference strains (Unemo et al., 2016). For the dose-fractionation experiments (*n* = 2), the activity of zoliflodacin was as follows: 1, 2, 3, and 4 g per day administered as the total dose given once, as one-half of the total dose given at 0 h and at 12 h (q12 h), and as one-third the total dose administered at 0, 8, and 16 h (q8 h). The dose-fractionation experiments were performed against the WHO X reference strain (Unemo et al., 2016).

### Quantification of Viable Bacterial Populations

To determine the total population and possible zoliflodacin-resistant subpopulations of *N. gonorrhoeae*, bacterial samples (1 ml) were withdrawn from the extra-capillary space of each cartridge at time points 3, 6.5, 24, 48, 72, 96, 120, 144, and 168 h for the dose-range experiments; at 3, 6.5, 12, 15, 18.5, 24, 48, 72, 96, 120, 144, and 168 h for q12 h dose-fractionation experiments; and at 3, 6.5, 8, 11, 14.5, 16, 19, 22.5, 24, 48, 72, 96, 120, 144, and 168 h for q8 h dose-fractionation experiments. Samples were serially diluted in mFB and quantitatively plated. Briefly, 100 µL of the diluted sample were plated onto GCAGP agar plates (Foerster et al., 2019) for quantification of the total bacterial population, and simultaneously, 100 µl were plated onto GCAGP agar plates (Foerster et al., 2019) containing 2–3 × MIC of zoliflodacin to identify potential zoliflodacin-resistant subpopulations. The zoliflodacin-free GCAGP agar plates (Foerster et al., 2019) were incubated for 24 h, and zoliflodacin-containing plates were incubated for up to 72 h at 36°C in a humidified 5% CO_2_-enriched atmosphere before the colony counts (log10 CFU/mL) were quantified using an automated colony counter (Scan 4000, Interscience, Saint-Nom-la-Bretèche, France).

### Zoliflodacin Concentration Determination

To confirm that the predicted PK profiles for zoliflodacin were observed in the HFIM, broth samples (500 µl) were collected at time points 1, 2, 3, 6.5, 18.5, 24, 48, 72, 96, 120, 144, and 168 h for the dose-range experiments; at 1, 2, 3, 6.5, 12, 15, 18.5, 24, 48, 72, 96, 120, 144, and 168 h for q12 h dose-fractionation experiments; and at 1, 2, 3, 6.5, 8, 11, 14.5, 16, 19, 22.5, 24, 48, 72, 96, 120, 144, and 168 h for q8 h dose-fractionation experiments. Samples were immediately frozen at −80°C until the zoliflodacin concentration was determined using liquid chromatography–tandem mass spectrometry (LC-MS/MS). Briefly, 100 µl sample/calibrator was pipetted into glass vials. Internal standard (10 µl of 25 mg/L cloxacillin sodium salt monohydrate (Vetranal, Sigma-Aldrich, Saint Louis, United States) in 18.2 MΩ ultrapure water) was added followed by mixing. Acetonitrile (150 μl; Gradient grade, LiChrosolv, Supelco) and 18.2 MΩ ultrapure water (750 µl) were added to each sample followed by mixing. Calibrators were prepared using the same nutrient liquid medium as was used in the HFIM experiment (mFB) and kept at −80°C until analysis (prepared at concentrations: 100, 10, 1, 0.1, and 0.02 mg/L). Samples were analyzed using LC-MS/MS. Instrumentation consisted of a Waters Acquity UPLC I-Class system with a binary pump and FTN injector fitted with a sample organizer. A Waters Xevo TQS-µ triple quadrupole mass spectrometer with electrospray was used for detection. Mass Lynx v 4.2 software was used for controlling the instrument and processing data. Injection volume was 1 µl, and the chromatographic column was an Acquity HSS T3 (1.8 µm 50*2.1 mm) column. A gradient with A: 0.1% formic acid in 18.2 MΩ ultrapure water and B: acetonitrile (flow rate 0.6 ml/min and a linear gradient running from 5%B to 100%B over 2 min) allowed co-elution of the analyte and its internal standard cloxacillin.

Within laboratory imprecision was estimated by analyzing five samples each on five days at three different concentrations. The coefficient of variation (CV) was calculated to 2.7% at 10 mg/L, 2.8% at 0.5 mg/L, and 12.7% at 0.025 mg/L. The low level of quantitation was set to 0.01 mg/L, showing within series CV of 8.0%. Linearity of response was determined over the range of 200–0.010 mg/L with 18 concentrations. Response was visually (as judged from residuals) as well as statistically curved, motivating a quadratic calibration function with 1/x weighting. Matrix effects were evaluated at two concentrations and found to be low (5% suppression at 0.1 mg/L and 1% suppression at 10 mg/L). With internal standard, a slight enhancement of signal response (analyte/internal standard) was observed at 4% at both low and high concentrations.

### Population Pharmacokinetic/Pharmacodynamic Mathematical Modeling

We simultaneously modeled 3 system outputs for the analysis of the experimental data. The system outputs were concentration of zoliflodacin, total *N. gonorrhoeae* burden, and burden of *N. gonorrhoeae* with decreased susceptibility/resistance to zoliflodacin (containing MIC-increasing *gyrB* target mutation selected during treatment). Population modeling was performed employing the nonparametric adaptive grid (NPAG) program of Leary et al. (2001) and Neely et al. (2012). Modeling choices (e.g., weighting) and goodness-of-fit evaluations were performed as previously published (Brown et al., 2015). Simulation was performed with the ADAPT 5 Program of D’Argenio et al. (2009) using Bayesian posterior parameter estimates.

To identify the dynamically linked PD index for *N. gonorrhoeae* cell kill, we chose to deconvolute the effect of zoliflodacin on the fully susceptible population vs. the preexistent less-susceptible population. This was done because in any of the arms where a resistant subpopulation amplification grew up, it would obscure the effect of zoliflodacin on the killing of the susceptible population. This was achieved by using the ADAPT 5 simulation routines with Bayesian parameter estimates only for the susceptible population. In addition, as we used the time-dependent effect on the susceptible population as a metric to identify the dynamically linked variable, we made all the susceptible population sizes the same. Also, because the values identified for clearance and volume were marginally different, we chose to average the identified values for these parameters for simulation of administration as a single dose once (full dose at 0 h), administration as q12 h (half the dose given at 0 h and half at 12 h), and q8 h (one-third of the dose at 0, 8, and 16 h).

### Whole Genome Sequencing

Whole genome sequencing (WGS) was performed, as previously described (Jacobsson et al., 2016; Golparian et al., 2020a), on selected colonies that grew on the zoliflodacin-containing plates and that also had increased zoliflodacin MIC when tested by agar dilution. The WGS was primarily performed to identify zoliflodacin resistance–associated *gyrB* mutations, that is, the previously identified *gyrB* mutations that were verified to cause the increased MICs of zoliflodacin in selected zoliflodacin-resistant mutants (Alm et al., 2015; Foerster et al., 2015; Foerster et al., 2019) or novel *gyrB* mutations. However, pairwise single-nucleotide polymorphism (SNP) analysis was also performed to compare the whole genome sequences of the previously sequenced WHO F and X reference strains and the corresponding selected zoliflodacin-resistant mutants. All reads were quality controlled and assembled using our customized CLC Genomics Workbench v20.0.4 workflow (Golparian et al., 2020b), and characterization of the full *gyrB* gene and identification of any novel or previously verified *gyrB* zoliflodacin resistance mutations (Alm et al., 2015; Foerster et al., 2015; Foerster et al., 2019) were obtained within the workflow using sequence mapping to *gyrB* of the reference strain FA1090 (GenBank: AE004969.1) and basic variant detection. Furthermore, reads for the identified *gyrB* mutants of WHO F and WHO X were mapped to the previously published (Unemo et al., 2016) reference genome sequence of WHO F (accession number: NZ_LT591897.1) and WHO X (accession number: NZ_LT592155.1), respectively, using Burrows–Wheeler Aligner (v0.7.17) with the MEM algorithm (Li et al., 2013) to identify additional mutations. Differences were identified using snp sites (v2.5.1) with default parameters (Page et al., 2016). Sequenced reads of WHO F *gyrB* mutants and WHO X *gyrB* mutants with increased MICs of zoliflodacin are available through the European Nucleotide Archive (ENA) accession number PRJEB44416.

## Results

### Phenotypic and Genetic Characteristics of Examined *N. gonorrhoeae* Strains

The zoliflodacin MICs determined using agar dilution and microbroth dilution, GyrB zoliflodacin resistance determinants, and additional relevant characteristics of the WHO F and WHO X reference strains are presented in Table 1.

**TABLE 1:** Relevant phenotypic and genetic characteristics of examined *Neisseria gonorrhoeae* strains.

Strain characteristics	WHO F, Unemo et al. (2016)	WHO X, Unemo et al. (2016)
Zoliflodacin agar dilution MIC (microbroth MIC)^a^	0.064 (0.125)	0.125 (0.25)
Ceftriaxone (MIC)^b^	<0.002	2
Cefixime (MIC)^b^	<0.016	4
Ciprofloxacin (MIC)^b^	0.004	>32
Azithromycin (MIC)^b^	0.125	0.5
GyrB codon D429, K450, S467	WT	WT
GyrA codon S91, D95	WT	S91F, D95N
ParC codon D86, S87, S88	WT	S87R, S88P
*mtrR* promoter region 13-bp inverted repeat	WT	Deletion of A
*mtrR* coding region	WT	WT
Mosaic *mtrRCDE*	—	—
PorB1b codon G120, A121	NA	G120K, A121D
NG-MAST	ST3303	ST4220
NG-STAR	ST2	ST226
MLST	ST10934	ST7363

MIC, minimum inhibitory concentration; WT, wild type; NA, not applicable; NG-MAST, *N. gonorrhoeae* multi-antigen sequence typing; ST, sequence type; NG-STAR, *N. gonorrhoeae* sequence typing antimicrobial resistance; MLST, multi-locus sequence typing.

a MIC (mg/L) was determined using agar dilution and microbroth methods for zoliflodacin.

b MIC (mg/L) was determined using Etest (bioMérieux, Marcy-l’Etoile, France) for ceftriaxone, cefixime, ciprofloxacin, and azithromycin.

Briefly, one *N. gonorrhoeae* reference strain that is susceptible to zoliflodacin and other modern antimicrobials (WHO F) and one XDR reference strain susceptible to zoliflodacin but resistant to ceftriaxone, cefixime, and ciprofloxacin (WHO X) were examined. The zoliflodacin MIC values were one MIC dilution higher when tested using the exploratory microbroth dilution method in mFB than by agar dilution on GCVIT agar plates.

### Hollow Fiber Infection Model Results

**FIGURE 1:**
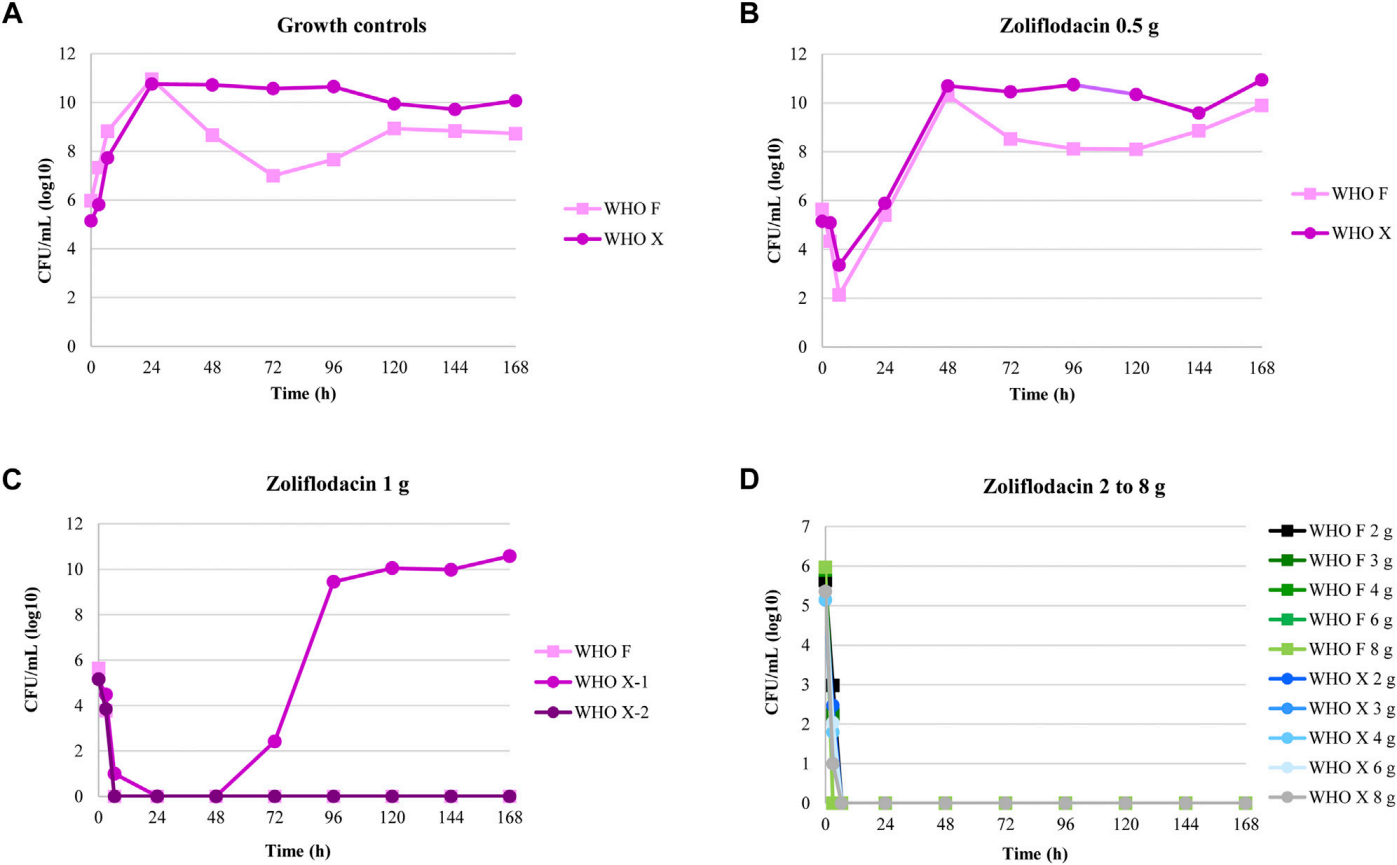
Growth curves of the total population of two *Neisseria gonorrhoeae* strains (WHO F and WHO X) in the dose-range hollow fiber infection model (HFIM) experiments (*n* = 2) simulating zoliflodacin single oral dose of 0.5, 1, 2, 3, 4, 6, and 8 g and followed for seven days. Notable, WHO X regrow after treatment with zoliflodacin 1 g in one of the two experiments.

The results of the zoliflodacin dose-range studies are summarized in Figure 1. Briefly, both examined strains grew well in the untreated growth control arms in the HFIM and reached a bacterial density of 10^10^–10^11^ CFU/ml at the 24-h time point. All untreated controls also maintained growth at approximately 10^9^–10^10^ CFU/ml throughout all 7-day experiments with a temporary decrease (reproducible in repeated experiments) in bacterial density for WHO F at 72-h time point (Figure 1A). A rapid bacterial kill was observed during the first 6.5 h for both strains with all zoliflodacin doses and experiments. Both strains regrew in the 0.5-g treatment arms, and after 24 h, the bacterial density was at approximately the same level as the initial inoculum. At 48 h, the bacterial concentrations were similar to those of the respective untreated controls (10^10^–10^11^ CFU/ml) (Figure 1B). In the 1-g treatment arms (Figure 1C), WHO F was rapidly killed in two experiments. However, for WHO X, the single-dose regimen of zoliflodacin 1 g eradicated this strain in the first experiment, while regrowth was observed after 72 h in the second experiment. Single doses of zoliflodacin 2–8 g reduced the bacterial densities to non-detectable concentrations after 6.5 h, and no strain regrew during any of the 7-day experiments (Figure 1D). Bacterial colonies were observed on zoliflodacin-containing agar plates for all experimental arms that showed regrowth. For the WHO F strain, treatment failure was observed for only the 0.5-g treatment arm. The colonies which grew on zoliflodacin-containing agar plates in this HFIM arm were a mixed population. Larger zoliflodacin-resistant colonies (zoliflodacin MIC = 0.5 mg/L using agar dilution) contained a G→A mutation in *gyrB* nucleotide position 1,285, encoding the GyrB D429N alteration, which fully explained the three-fold increase in the zoliflodacin MIC (Alm et al., 2015; Foerster et al., 2015; Foerster et al., 2019). Compared to the WHO F reference genome (Unemo et al., 2016), these zoliflodacin-resistant *gyrB* mutants contained the GyrB D429N alteration, but also occasional SNPs in or associated with the multicopy *opa* and pili genes. However, the SNPs in the *opa* and pili genes did not appear to affect the MICs of zoliflodacin, and they may also reflect the limitations of short sequence reads in the mapping to multicopy genes. Smaller zoliflodacin-susceptible colonies (MIC = 0.125 mg/L using agar dilution) lacking GyrB alterations were also found. These zoliflodacin-susceptible colonies reflected that when WHO F grew at very high-density cell concentrations in the HFIM cartridges, rare colonies managed to survive especially on the 2 × MIC zoliflodacin-containing agar plates. This also shows the importance of always performing antimicrobial MIC testing as well as WGS of all suspected resistant mutants on the antimicrobial-containing agar plates in HFIM experiments. For the WHO X strain, treatment failure was observed for both experiments for the single-dose zoliflodacin regimen of 0.5 g and in one of two experiments for the single-dose regimen of 1 g. The WHO X colonies which grew on zoliflodacin-containing agar plates from both regimens had zoliflodacin MICs of 0.5–1 mg/L (agar dilution) compared to a zoliflodacin MIC of 0.125 mg/L (agar dilution) for the parent isolate. These colonies harbored the GyrB D429N alteration, which explained the two–three fold increase in the zoliflodacin MIC. Occasional SNPs in the multicopy *opa* genes were also detected in the WHO X *gyrB* mutants, when comparing to the WHO X reference genome (Unemo et al., 2016). These SNPs did not appear to affect the MICs of zoliflodacin, and, again, they may reflect the limitations of short sequence reads in the mapping to multicopy genes.

**FIGURE 2:**
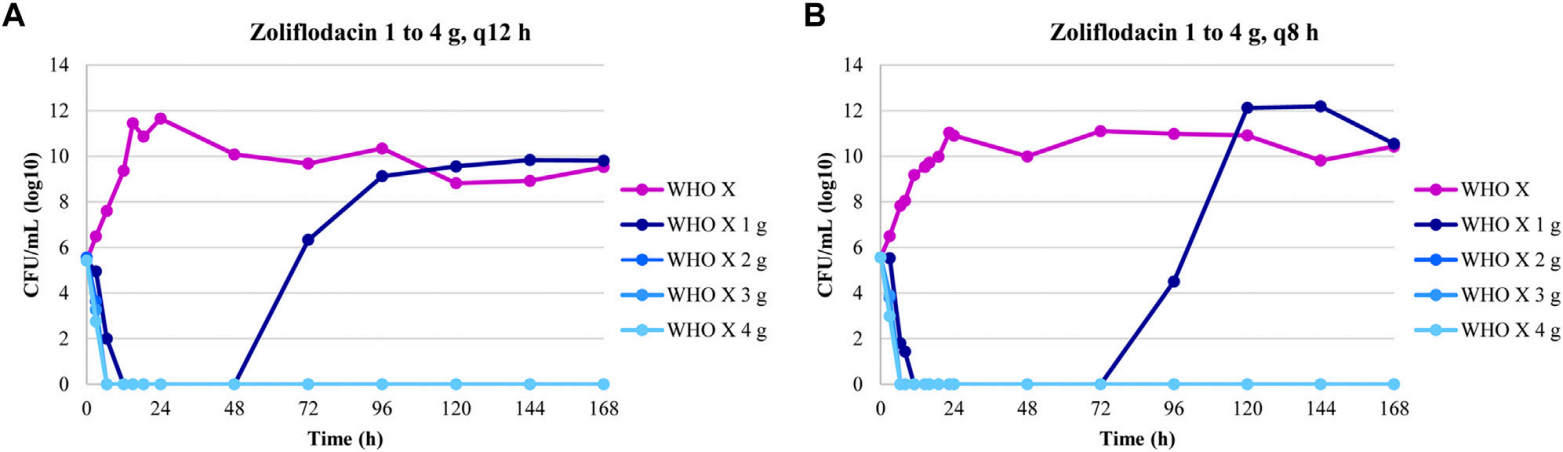
Growth curves of the total population of the *Neisseria gonorrhoeae* WHO X reference strain in the dose-fractionation hollow fiber infection model (HFIM) experiments (*n* = 2) simulating zoliflodacin single oral dose of 1, 2, 3, and 4 g administered as equally divided doses at q12 h and q8 h over 24 h and followed for 7 days.

The dose-fractionation experiments simulating a single 1, 2, 3, and 4 g oral dose of zoliflodacin administered q12 h and q8 h for 24 h against WHO X showed similar growth and sterilization patterns in the different treatment arms as in the dose-range experiments. As with the 1 g dose given once, also the 1 g dosage given as equally divided doses at q12 h and q8 h for 24 h failed to eradicate WHO X (Figure 2). The WHO X zoliflodacin-resistant mutants that resulted in regrowth in these failed treatment arms had an MIC of 0.5 mg/L using agar dilution.

### Population Pharmacokinetic/Pharmacodynamic Modeling

The three-output PK/PD model was fit to all the data separately, for both WHO F and WHO X. The mean and median values for WHO F and WHO X, respectively, are displayed in Table 2.

**TABLE 2:** Mean, median, and standard deviation of the parameter values for the hollow fiber infection model (HFIM) study with *Neisseria gonorrhoeae* reference strains WHO F (WHO X).

Parameter	Mean	Median	Standard deviation
V_c_ (L)	1,076 (1,066)	1,022 (1,081)	65.16 (274.4)
CL (L/hr)	116.7 (119.2)	105.6 (116.6)	13.11 (28.12)
k_g-s_ (hr^−1^)	1.142 (1.163)	1.086 (1.407)	0.07051 (0.4059)
k_g-r_ (hr^−1)^	0.5602 (1.206)	0.5987 (1.680)	0.06005 (0.9231)
K_kill-s_ (hr^−1^)	4.524 (20.74)	4.722 (18.11)	0.2418 (5.846)
K_kill-r_ (hr^−1^)	1.519 (3.256)	1.502 (3.661)	0.03657 (1.374)
C_50-s_ (mg/L)	0.2507 (0.7454)	0.2885 (0.6349)	0.04692 (0.3133)
C_50-r_ (mg/L)	0.4334 (1.520)	0.4491 (1.059)	0.03111 (1.276)
H_s_ (---)	1.581 (8.494)	1.490 (4.963)	0.2066 (5.870)
H_r_ (---)	4.377 (11.68)	4.013 (13.07)	0.7291 (5.976)
POPMAX (CFU/ml)	5.981 × 10^9^ (2.665 × 10^11^)	9.913 × 10^9^ (9.149 × 10^10^)	4.601 × 10^9^ (2.896 × 10^11^)
IC2 (CFU/ml)	6.723 × 10^5^ (2.922 × 10^5^)	7.851 × 10^5^ (2.471 × 10^5^)	1.535 × 10^5^ (2.352 × 10^5^)
IC3 (CFU/ml)	6.405 (8.080)	9.912 (5.478)	4.143 (7.135)

V_c_, apparent volume of the central compartment; CL, clearance; K_g-s_ and K_g-r_, rate constants of growth for the susceptible and resistant populations, respectively; K_kill-s_ and K_kill-r_, rate constants of kill for the susceptible and resistant populations, respectively; C_50-s_ and C_50-r_, concentrations of zoliflodacin at which the kill rate is half maximal for the susceptible and resistant populations, respectively; H_s_ and H_r_, Hill’s constants for the susceptible and resistant populations, respectively (unitless); POPMAX, maximal population size; CFU, colony-forming unit; IC2 and IC3, sizes of the total and resistant populations, respectively, at therapy initiation.

**FIGURE 3:**
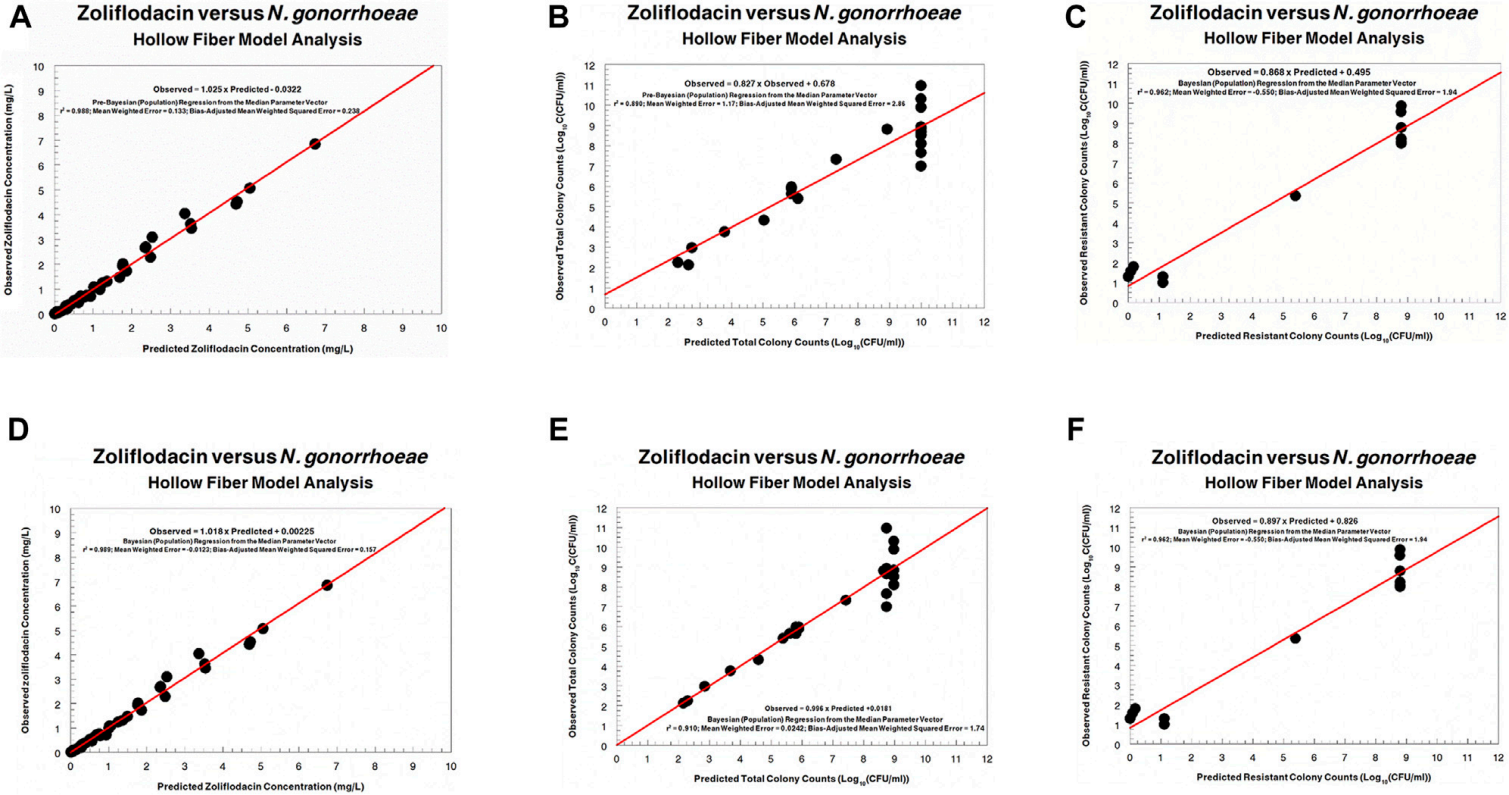
Predicted–observed regressions for zoliflodacin concentrations, total *Neisseria gonorrhoeae* burden, and resistant *N. gonorrhoeae* burden for the pre-Bayesian regression **(A–C)** and for the Bayesian regressions **(D–F)** for WHO F.

**FIGURE 4:**
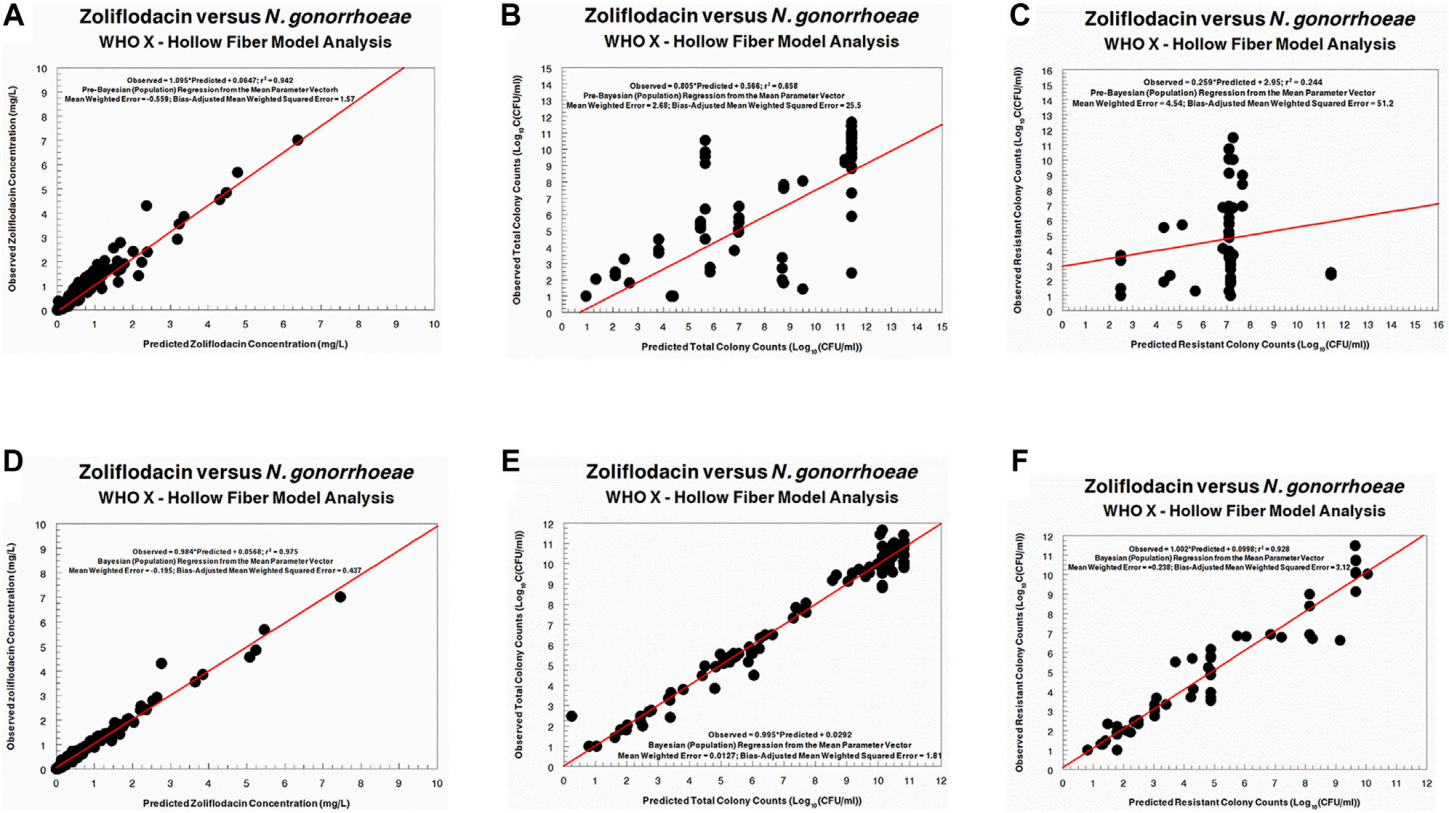
Predicted-observed regressions for zoliflodacin concentrations, total *Neisseria gonorrhoeae* burden, and resistant *N. gonorrhoeae* burden for the pre-Bayesian regression **(A–C)** and for the Bayesian regressions **(D–F)** for WHO X.

The fit of the model to the data was acceptable. The predicted–observed regressions for both analyses are displayed in Figures 3, 4. In both, panels A–C are the pre-Bayesian (population) regressions, and panels D–F are the Bayesian (individual) regressions.

Regarding the zoliflodacin PK profiles, the agreement between observed and predicted zoliflodacin concentrations in the HFIM during the experiments was high (Figures 3A,D, 4A,D).

The HFIM study for the *N. gonorrhoeae* WHO F strain was a dose-ranging study (Figure 1). There was no system information to allow identification of the dynamically linked PD indices for cell kill and zoliflodacin resistance suppression. Nonetheless, we performed simulation with the identified parameter values to obtain an approximate exposure of zoliflodacin that would suppress amplification of mutants with increased zoliflodacin MIC and *gyrB* resistance mutations. That exposure corresponded to an AUC/MIC ratio (concentration dependency) of 70.6 (using the microbroth dilution MIC value).

The HFIM study for the *N. gonorrhoeae* WHO X strain had full dose fractionation for administration of zoliflodacin doses of 1, 2, 3 and 4 g once, twice (half of the dose given, separated by 12 h), and three times (one-third of the dose given, separated by 8 h) (Figure 2). As we wished to link a mode of administration to the most efficient *N. gonorrhoeae* kill, we performed simulation for each of the doses employing the Bayesian parameter estimates for that dose and administration schedule (available from the authors upon request). Resistance emergence would confound the effect of zoliflodacin on the susceptible population, so for the linkage to bacterial cell kill, we deconvoluted the effect on the susceptible population from the effect on the total (susceptible plus mutants with increased zoliflodacin MIC) population. In addition, we employed the average value of volume and clearance (Bayesian) for each dose, as we wished to directly compare the impact of schedule. In like manner, we fixed the baseline susceptible burden to 10^6^ CFU/ml. Notably, the kill-rate constants for both the susceptible and resistant populations, as well as zoliflodacin concentrations at which the kill rate is half maximal, were substantially higher for WHO X than WHO F (Table 2). The kill-rate constants for resistant populations were approximately 2.7 times higher than the growth rate constants for both strains (Table 2). However, WHO X showed a more effective kill of the susceptible population, that is, the kill rate constant was nearly 18 times higher than the growth rate constant. For comparison, this value was only approximately 4 times higher for WHO F (Table 2).

**FIGURE 5:**
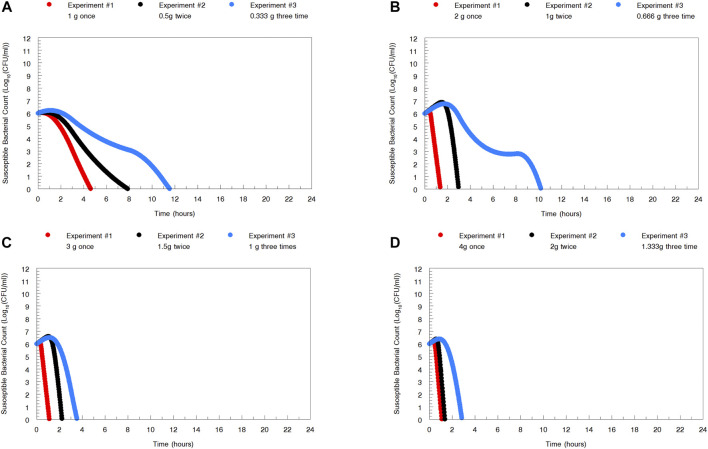
To examine the impact of administration schedule on the rate of *Neisseria gonorrhoeae* WHO X kill, simulations were performed for the whole zoliflodacin dose administered at once, half the dose twice 12 h apart, and one-third the dose three times 8 h apart. The most rapid *N. gonorrhoeae* kill was obtained by administering the full dose once. However, for larger doses, that is, over 2 g, of zoliflodacin, the schedule of doses becomes less relevant.

In Figure 5, we display the impact of administration schedule on the rate of *N. gonorrhoeae* cell kill. There are differences by dose, as would be expected, but there are clear differences also by schedule of administration, with the most rapid decline in CFU counts being obtained by administering the full dose once. The difference is clearest for the 1 and 2 g total doses. For larger doses, that is, over 2 g, the schedule of doses becomes less relevant. This is to be expected as there is a maximal kill-rate constant identified as part of the modeling process.

In examining Figure 2, the zoliflodacin 1 g dose failed when given fractionated in 2 or 3 parts (q12 and q8 h). When given all at once, it failed in one experiment but succeeded in the other (Figure 1). This indicates that administration of the whole dose all at once may also be best for resistance suppression. However, more data before fully accepting this hypothesis would be beneficial.

## Discussion

Antimicrobial resistance in *N. gonorrhoeae* is compromising the treatment and control of gonorrhea globally, and decreased susceptibility or resistance to the last option for empiric first-line treatment, ceftriaxone, has been reported worldwide (Wi et al., 2017; Unemo et al., 2019). The first-in-class spiropyrimidinetrione, zoliflodacin, has in previous studies shown to be bactericidal, to have a low frequency of resistance, and to have high *in vitro* activity against *N. gonorrhoeae*, including multidrug-resistant and XDR strains (Alm et al., 2015; Basarab et al., 2015; Foerster et al., 2015; Foerster et al., 2019; Bradford et al., 2020; Unemo et al., 2021). Furthermore, in a phase 2 RCT therapy with a single dose of zoliflodacin 3 g, it was shown to microbiologically cure all urogenital (47/47) and rectal (6/6), and 78% (7/9) of pharyngeal infections according to the per-protocol analyses The corresponding cure rates with a single dose of zoliflodacin 2 g were 98% (48/49), 100% (4/4), and 67% (4/6), respectively (Taylor et al., 2018). Of note, the number of extragenital infections was small. However, despite these clinical treatment failures, no *N. gonorrhoeae* isolates with *in vitro* resistance to zoliflodacin were found (Taylor et al., 2018). Unfortunately, the lack of appropriate ways to model PK/PD in a dynamic system mimicking the infection has previously hindered a better understanding of the findings of the zoliflodacin phase 2 RCT (Taylor et al., 2018) and the optimization of zoliflodacin dosing regimens for ideal *N. gonorrhoeae* kill as well as suppression of resistance emergence.

In the present study using our dynamic *in vitro* HFIM, we show that to provide effective *N. gonorrhoeae* killing and resistance suppression, zoliflodacin should ideally be administered as a sufficiently large single dose. We also show that the activity of zoliflodacin is mainly concentration-dependent rather than time-dependent (Figure 5). The mechanisms causing concentration-dependency vs. time-dependency and the clinical implications and relevance of distinguishing between these two have been previously elucidated in detail (Drusano, 2004). Our results indicate that for larger zoliflodacin doses, that is, over 2 g, the administration schedule became less relevant, because the rate of *N. gonorrhoeae* kill was approaching a maximum. Moreover, in the clinic, administration of a single dose provides the additional advantage of no concerns regarding adherence with subsequent doses. In general, zoliflodacin had high kill-rate constants compared to the growth-rate constants that resulted in a rapid decline of *N. gonorrhoeae* CFUs. However, single-dose oral treatment with 0.5 g failed to eradicate both examined gonococcal strains, and also 1 g single dose failed in one of two experiments for the WHO X strain, which was also observed for 1 g treatment given as equally divided doses at q12 h and q8 h over 24 h. These failures were caused by the suboptimal zoliflodacin doses and insufficient exposure to suppress emergence of zoliflodacin-resistant populations containing a GyrB D429N alteration, which previously has been verified to cause this level of zoliflodacin resistance *in vitro* (Alm et al., 2015; Foerster et al., 2015; Foerster et al., 2019). In previous static time-kill experiments over 1–3 days, resistance to zoliflodacin was difficult to select (Alm et al., 2015; Foerster et al., 2015; Foerster et al., 2019). However, using the dynamic HFIM and experiments over 7 days, zoliflodacin resistance amplification was observed from 24-h time point and onward for all doses failing to eradicate the WHO F and/or WHO X strains (single oral zoliflodacin dose of 0.5 and 1 g; and 1 g given as equally divided doses at q12 h and q8 h over 24 h). Based on the present HFIM results examining zoliflodacin-susceptible gonococcal strains, a single oral dose of ≥2 g zoliflodacin should be effective in treating most gonococcal infections as well as suppress resistance emergence*.* A single oral dose of zoliflodacin 2 g resulted in zoliflodacin exposure corresponding to AUC/MIC ratio (concentration-dependency) of 137.6 and 75.6 for WHO F and WHO X, respectively (using the microbroth dilution MIC values). Based on all available data, a single oral 3 g dose of zoliflodacin is the appropriate dose to study in the phase 3 RCT for treatment of uncomplicated gonorrhea (ClinicalTrials.gov identifier NCT03959527), that is, considering also that no clinical strains with *gyrB* target resistance mutations or other potentially important *gyrB* mutations affecting the zoliflodacin susceptibility have been examined in the HFIM, only PK data for free (protein-unbound) zoliflodacin concentrations in human plasma can be used to design this type of PK/PD studies (because no zoliflodacin PK data for gonococcal infection sites are available), and to provide a safety margin to cover gonococcal and human interpopulation variance, which can substantially affect kill efficacy and resistance suppression of an antimicrobial (Drusano, 2004). This is also in line with the results of the zoliflodacin phase 2 RCT for gonorrhea, where an increased number of treatment failures were observed with zoliflodacin 2 g single oral dose (Taylor et al., 2018). No *N. gonorrhoeae* isolates with *in vitro* resistance to zoliflodacin were found (Taylor et al., 2018), and the failures to cure were accordingly likely not due to bacterial resistance or reinfection but because of an insufficient exposure of zoliflodacin, especially in the pharynx where nearly all treatment failures were observed both with zoliflodacin 2 and 3 g doses. If approved for wide use in the clinic, the risk for selection of zoliflodacin-resistant *N. gonorrhoeae* strains may emerge. Therefore, the use of zoliflodacin treatment regimens able to ensure effective *N. gonorrhoeae* killing as well as suppression of resistance emergence is imperative. Accordingly, it is crucial to carry out studies providing zoliflodacin PK data for the urogenital and extragenital infection sites, particularly in the pharynx, and additionally to examine treatment of gonococcal strains with different relevant *gyrB* mutations. Finally, evaluation of zoliflodacin in HFIM antimicrobial combination studies to ensure effective eradication of all gonococcal strains, suppress resistance emergence, and potentially cure concomitant STIs merit considerations.

The main limitation of this study is the absence of zoliflodacin PK data from gonorrhea infection sites, such as the anogenital tract and the oropharynx. Consequently, our HFIM simulations had to be based on concentrations of free zoliflodacin in human plasma which may not ideally reflect systemic exposures achieved at urogenital and extragenital infection sites. However, antimicrobial concentrations in serum or plasma are frequently used as a surrogate for antimicrobial concentrations at the infection sites for many bacterial species (because of the lack of known infection site concentrations), and in most cases, these surrogates serve relatively well in the aim to link drug exposure to effect (Drusano. 2004). For gonorrhea and other STIs, the lack of adequate PK data from infection sites, especially the oropharynx, is unfortunately a general problem affecting current gonorrhea therapeutic antimicrobials as well as those in development (Kong et al., 2019). Accordingly, such PK data are imperative to generate, and PK studies should ideally be included in all RCTs for the treatment of gonorrhea and other STIs. In addition, research is required for further understanding of pharyngeal gonorrhea and where and how to measure the relevant PK parameters of gonorrhea therapeutic antimicrobials in the pharynx/mouth, and their significance (Kong et al., 2019). It is also imperative to study inter-patient variance in PK parameters from population modeling and employing these data in Monte Carlo simulations for target attainment (Drusano et al., 2001; Drusano, 2004).

In conclusion, by examining the pharmacodynamics of zoliflodacin against *N. gonorrhoeae* in our dynamic *in vitro* HFIM for gonorrhea, we show that to provide both effective *N. gonorrhoeae* killing and resistance suppression, zoliflodacin should ideally be administered as a sufficiently large single dose. We also demonstrated a rapid bacterial kill during the first 6.5 h for both examined WHO *N. gonorrhoeae* reference strains, all zoliflodacin doses, and in all experiments. However, zoliflodacin resistance amplification was observed for all doses failing to eradicate the examined strains (single oral zoliflodacin dose of 0.5 and 1 g; and 1 g given as equally divided doses at q12 h and q8 h for 24 h). Considering that no clinical strains with *gyrB* mutations affecting the zoliflodacin susceptibility have been examined in the HFIM, only zoliflodacin PK data for human plasma can be used to design this type of PK/PD studies, and to maintain a safe efficacious exposure to cover gonococcal and human interpopulation variance, our data support the use of a single oral 3 g dose of zoliflodacin in the phase 3 RCT for treatment of uncomplicated gonorrhea. Thus, the present study not only provides further understanding of the findings of the zoliflodacin phase 2 RCT (Taylor et al., 2018), the concentration-dependent killing of *N. gonorrhoeae* with zoliflodacin (most effective when giving the whole dose once), importance of examining multiple divergent *N. gonorrhoeae* strains, and suppression of AMR emergence but also serves us to inform the initiated zoliflodacin phase 3 RCT (ClinicalTrials.gov identifier NCT03959527) as well as future dosing, registration, and introduction of zoliflodacin in clinical practice for gonorrhea. However, to further optimize the accuracy of dose prediction, studies providing zoliflodacin PK data for the urogenital and extragenital infection sites, particularly in the pharynx, and examining treatment of gonococcal strains with different relevant *gyrB* mutations would be important.

## Data Availability

The datasets presented in this study can be found in online repositories. The names of the repository/repositories and accession number(s) can be found below: European Nucleotide Archive accession number PRJEB44416.
